# The dose distribution in dominant intraprostatic tumour lesions defined by multiparametric MRI and PSMA PET/CT correlates with the outcome in patients treated with primary radiation therapy for prostate cancer

**DOI:** 10.1186/s13014-018-1014-1

**Published:** 2018-04-12

**Authors:** Constantinos Zamboglou, Christina Marie Klein, Benedikt Thomann, Thomas Franz Fassbender, Hans C. Rischke, Simon Kirste, Karl Henne, Natalja Volegova-Neher, Michael Bock, Mathias Langer, Philipp T. Meyer, Dimos Baltas, Anca L. Grosu

**Affiliations:** 1 Department of Radiation Oncology, Medical Center – University of Freiburg, Faculty of Medicine, University of Freiburg, Robert-Koch Straße 3, 79106 Freiburg, Germany; 2 Division of Medical Physics, Department of Radiation Oncology, Medical Center – University of Freiburg, Faculty of Medicine, University of Freiburg, Freiburg, Germany; 3 Department of Nuclear Medicine, Medical Center – University of Freiburg, Faculty of Medicine, University of Freiburg, Freiburg, Germany; 4 Department of Radiology, Medical Center – University of Freiburg, Faculty of Medicine, University of Freiburg, Freiburg, Germany; 5 German Cancer Consortium (DKTK), Partner Site Freiburg, Freiburg, Germany; 6 Berta-Ottenstein-Programme, Faculty of Medicine, University of Freiburg, Freiburg, Germany

**Keywords:** Prostate cancer, MRI, PSMA PET/CT, Dominant lesion, Radiation dose

## Abstract

**Background:**

We hypothesized that dominant intraprostatic lesions (DILs) could be depictured by multimodal imaging techniques (MRI and/or PSMA PET/CT) in patients with primary prostate cancer (PCa) and investigated possible effects of radiotherapy (RT) dose distribution within the DILs on the patients’ outcome.

**Methods:**

One hundred thirty-eight patients with localized prostate cancer (PCa) and visible DIL underwent primary external beam RT between 2008 and 2016 with an aimed prescription dose of 76 Gy to the whole prostate. Seventy-five patients (54%) additionally received androgen deprivation therapy. Three volumes were retrospectively generated: DIL using pretreatment MRI and/or PSMA PET/CT, prostatic gland (PG) and the subtraction between PG and DIL (SPG). The minimum dose (Dmin), maximum dose (Dmax) and mean dose (Dmean) in the three respective volumes were calculated. Biochemical recurrence free survival (BRFS) was considered in uni- and multivariate Cox regression analyses. An explorative analysis was performed to determine cut-off values for the three dose parameters in the three respective volumes.

**Results:**

With a median follow-up of 45 months (14–116 months) 15.9% of patients experienced BR. Dmin (cut-off: 70.6 Gy, HR = 0.39, *p* = 0.036) applied to the DIL had an impact on BRFS in multivariate analysis, in contrast to the Dmin delivered to PG and SPG which had no significant impact (*p* > 0.05). Dmin was significantly (*p* < 0.004) lower in patients with BR than in patients without BR. Dmax within DIL-imaging (cut-off: 75.8 Gy, HR = 0.31, *p* = 0.009) and in both PG und SPG (cut-off: 76 Gy, HR = 0.32, *p* = 0.009) had a significant impact on the BRFS. 95% of patients with a Dmax ≥76 Gy in SPG had a Dmin ≥70.6 Gy in DIL-imaging. Dmean in all of the three volumes had no significant impact on BRFS (*p* > 0.05).

**Conclusions:**

The dose distribution within DILs defined by PSMA PET/CT and/or MRI is an independent risk factor for BR after primary RT in patients with PCa. These findings support the implementation of imaging based DIL interpretation for RT treatment planning, although further validation in larger patient cohorts with longer follow-up is needed.

## Background

Prostate cancer (PCa) is known to be a multifocal disease [1]. Likewise, conventional external beam radiation therapy (EBRT) for patients with primary PCa aims at delivering a homogeneous dose to the entire prostatic gland. However, there is growing evidence that dominant intraprostatic lesions (DIL) within the gland may be responsible for metastatic and recurrent disease. Haffner et al. tracked the clonal origin in a patient who died of metastases from PCa and proved that all metastases arose from a single prostatic lesion [2]. Three studies examined whether the local recurrences of PCa after primary radiation therapy (RT) occur at the site of primary lesion using pre and post treatment magnetic resonance imaging (MRI) in a limited number of patients, respectively. All of them concluded that local recurrence after RT occurs mostly at the side of the primary tumour [3–5]. However, our group and others performed comparison studies between MRI, prostate-specific membran antigen positron emission tomography/computed tomography (PSMA PET/CT) and PCa in surgery specimen. Sensitivities of 52–85% for MRI [6] and 64–75% for PSMA PET/CT [7–9] were reported, suggesting, that not the entire PCa amount is identified by these techniques mainly because of non-detectable microscopic lesions.

A dose-response relationship between RT dose to the entire prostatic gland and PCa control rates has been reported. A meta-analysis demonstrated, that the total RT dose on the prostatic gland reduces the risk of biochemical failure in patients with primary PCa by approximately 1.8% for each 1-Gray (Gy) increase [10]. Martinez et al. reported a significant decrease in biochemical failures when a biological equivalent dose BED_α/β = 1.2 Gy_ > 268 Gy was delivered to the prostate by a combination of EBRT and high-dose rate brachytherapy [11]. There is limited evidence [12] if the RT dose delivered to the imaging defined PCa has an impact on the tumour control.

In this analysis we hypothesized that the DIL could be depictured by multimodal imaging techniques: multiparametric MRI (mpMRI) and/or PSMA PET/CT. Likewise, we tested whether the RT doses delivered to the DILs may influence the patients’ outcome.

## Methods

### Patients

This retrospective, single institution analysis enrolled patients with localized and histologically proven PCa who received EBRT with or without androgen deprivation therapy (ADT) from February 2008 to October 2016. The availability of mpMRI images or PSMA PET/CT scans at the maximum of 6 months prior to EBRT was mandatory. Patients were excluded from the analysis if they received EBRT of the pelvic lymph nodes, had cN1 or cM1 disease, had initial prostate specific antigen (PSA) serum values above 50 ng/ml or had no detectable intraprostatic lesion in PET and mpMRI. This study was approved by the institutional review board.

### PSMA PET/CT and MRI imaging

MR images were acquired either on a 3 Tesla or on a 1.5 Tesla system. All systems were equipped with a surface phased array in combination with an integrated spine array coil. No endo-rectal coil was used. Essentially, T2-weighted fast spin echo (T2w-TSE) images, diffusion weighted images (DWI) and dynamic contrast-enhanced (DCE) perfusion images were acquired. A detailed description of the MR imaging protocol is given in [13]. In case of multiple mpMRI scans before the treatment the last scan prior RT was selected for analysis.

Radiolabelled tracers targeting the prostate specific membrane antigen (PSMA) have been used for detection and delineation of intraprostatic tumour. PET/CT scans were performed one hour after injection of the ligand ^68^Ga-HBED-CC-PSMA [14] with a 64-slice GEMINI TF PET/CT or a 16-slice GEMINI TF BIG BORE PET/CT (both Philips Healthcare. USA). Both imaging systems were cross-calibrated. A detailed description of our ^68^Ga-HBED-CC-PSMA PET/CT imaging protocol is given in [13].

Prior to EBRT 131 (95%) patients received mpMRI and 36 (26%) patients received PSMA PET/CT scans. Twenty-nine patients (21%) had both.

### Image co-registration and generation of contours

Axial T2w and/or CT (derived from PET/CT scans) images were matched with the planning CT in the RT planning system Eclipse v13.5 (Varian, USA) using mutual information registration. In case visual assessment showed an anatomical mismatch, a manual adjustment was performed based on anatomical markers. The usage of an axial T2w sequence and at least one DWI- or DCE sequence was mandatory. When available from the DWI data, the calculated apparent diffusion coefficient (ADC) maps were registered (84 patients, 61%), whereas from the DCE time series a post-injection time frame was manually selected for having a maximum contrast in the prostate (113 patients, 82%). For further alignment between PSMA PET and CT images and T2w images and the DWI- or DCE sequences the respective pre-set registrations were used.

**Fig. 1:**
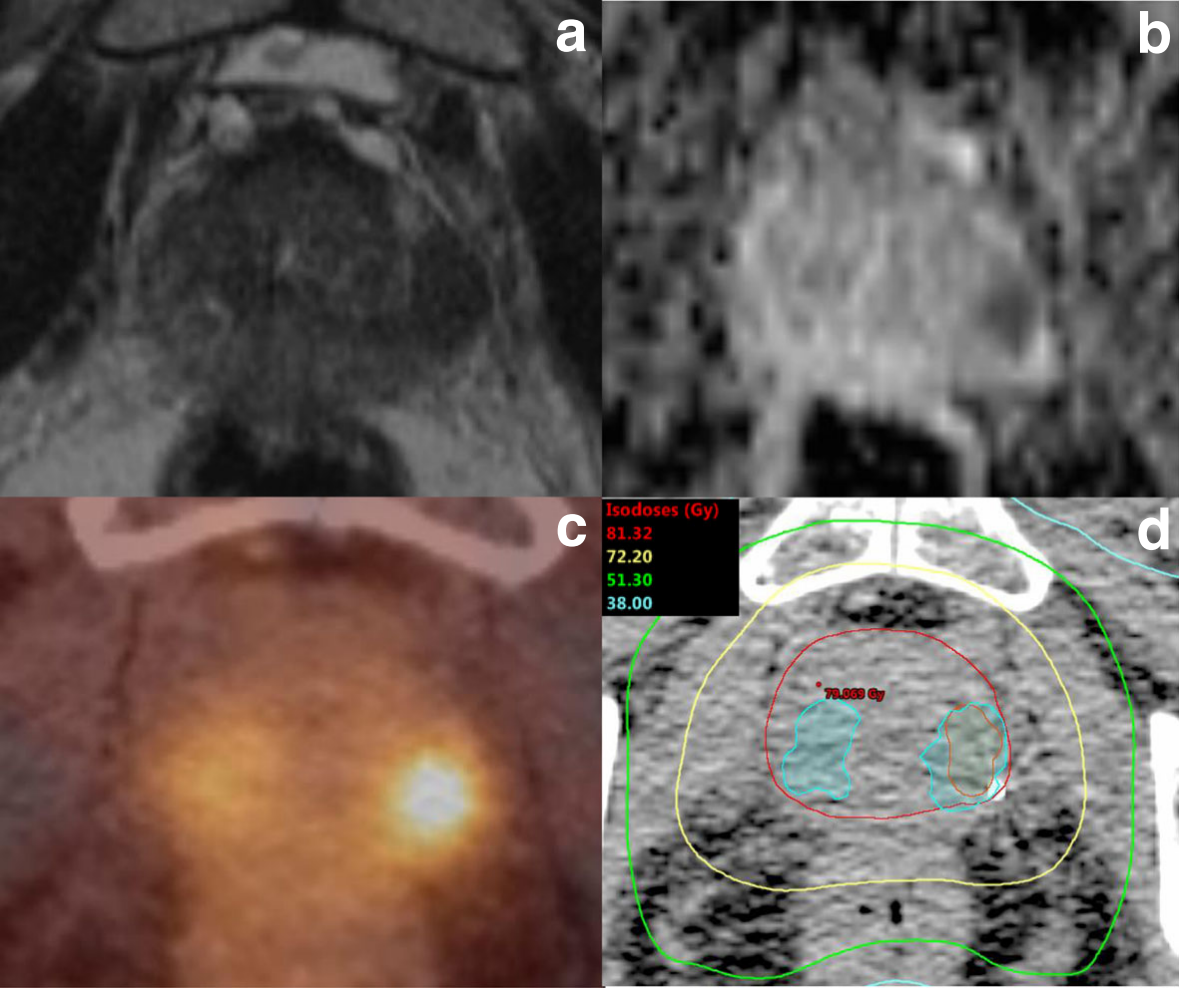
Correlation between tumor volume depicted in multimodal imaging and dose distribution. A 82 year old patient with biopsy confirmed PCa (Gleason score 9) and an initial PSA of 8 ng/ml underwent mpMRI (**a**:T2w, **b**:ADC), PSMA PET/CT (**c**) and a planning CT (**d**) before EBRT. MpMRI depicted one lesion in the left lobe and PSMA PET depicted one lesion in the left lobe and one lesion in the right lobe. In picture D the IMRT dose-distribution with contours of the prostatic gland (red), PSMA PET (blue) and MRI (orange) are shown. Dmax (red dot) was located outside the DIL-imaging volume

Contouring was performed in Eclipse v13.5. The gross tumour volume according to PET information (GTV-PET) was created semi-automatically using a threshold of 30% of the maximum standardized uptake value (SUVmax) within the prostate which was derived from a previous study by our group [15]. Two experienced readers delineated GTV based on mpMRI (GTV-MRI) in consensus using T2W, DWI and DCE-sequences to characterize each lesion. Only lesions with visually determined “Prostate Imaging - Reporting and Data System Version 2” (PI-RADs v2) [16] score 4 or higher were included in the analysis. In cases with both PET and mpMRI information (29 patients), GTV-PET and GTV-MRI were combined to GTV-union which was used for further analyses in the respective patients (Fig. 1). In the following GTV-MRI, GTV-PET and GTV-union are summarized as DIL-imaging. The prostatic gland (PG) was delineated using the CT and T2w-MRI information (if available) by two experienced readers in consensus. In case of seminal vesicle involvement (9 patients) the parts of the seminal vesicles containing PCa (therefore part of DIL-volume) were also added to the whole PG volume. To define the non-PCa prostate tissue (SPG), DIL-imaging was subtracted from PG.

### Treatment protocol

Patients were advised to have a full bladder and empty rectum during the whole treatment. Planning computed tomography (CT) was acquired in supine position. RT was delivered in 41 (30%) and 97 (70%) patients with 3D-conformal and intensity-modulated RT (IMRT), respectively. All patients had image-guided RT (IGRT) using daily 2D/2D imaging and at least one cone-beam CT (cbCT) per week. Intraprostatic fiducial markers were implanted in 130 (94%) patients prior to EBRT. Using the cbCT information the contours of the organs at risk as well as the target volumes were adapted in the IMRT group. Taking into account D’Amico’s risk stratification [17] the clinical target volume (CTV) was defined as the prostatic gland ±4 mm ± the base, half or the entire seminal vesicles, considering the rectal wall as anatomical border. The CTV was expanded with 6 mm to create the planning target volume (PTV).

The aimed prescription dose was 76 Gy to the entire prostatic gland. No RT dose escalation to intraprostatic volumes was performed. In our cohort, the median applied dose to the PTV was 74 Gy (range: 66–78 Gy) delivered for 14 patients in fractions of 1.8 Gy and 2 Gy for the remaining 124 patients. Seventy-five patients (54%) received ADT parallel to EBRT for a median duration of 7 months (range: 3–24 months).

During follow-up patients were seen every 3–6 months for the first 2 years and every 6–24 months thereafter for physical examination and PSA measurements. Follow-up examinations were performed at our institution or from another board-licensed urologist. Radiologic evaluation by MRI, CT or PET/CT (PSMA or choline) was conducted if clinically indicated.

### Data analysis and statistics

Biochemical recurrence (BR) after EBRT according to the Phoenix criteria [18] was defined as the study endpoint. The dose information, including mean dose (Dmean), minimum dose (Dmin) and maximum dose (Dmax) were calculated in PG, SPG and DIL-imaging, respectively, using the dose volume histograms (DVHs) of the RT treatment plans of each patient. Due to limited knowledge about dose distributions in the respective volumes and their correlation with BR, we performed an explorative analysis to determine cut-off dose values for further calculations: the respective median Dmin, Dmax and Dmean values for all volumes in the entire group were calculated. Considering these values (lowest median value minus 2 Gy and highest median value plus 2 Gy) the ranges for the analysis were defined: Dmin: 70–75.2 Gy, Dmax: 75–79.8 Gy, Dmean: 73.5–77.3 Gy. Univariate Cox regression analyses were performed for each dose parameter in 0.1 Gy steps for PG, SPG and DIL-imaging, respectively. For each of the three volumes the significant dose parameter with the lowest hazard ratio (HR) was used for further analyses provided that at least 20 patients were analyzed per group.

Multivariate Cox regression analyses adjusted for clinical T stage and Gleason score (significant in Cox regression analyses including patient related parameters) were performed analyzing the impact of the respective dose parameters on BR free survival (BRFS).

For the graphical representation the respective dose parameters were analyzed by Kaplan-Meier survival curve compared by log-rank test.

All tests were considered to be statistically significant at *p* < 0.05. Statistical analysis was conducted with SPSS v22 (IBM, USA).

## Results

### Patient and treatment characteristics

One hundred thirty-eight patients were included into the analysis. The median age of patients was 74 years (range: 56–85 years). The majority of patients (75%) had high-risk disease according to the D’Amico classification [17]. The detailed characteristics of the study cohort are listed in Additional file 1: Table S1. In univariate analysis with patient related parameters Gleason score, volume of DIL-imaging and cT stage were significant for BRFS (Table 2). In multivariate analysis including the significant patient related parameters only cT stage and Gleason score remained significant (*p* < 0.05).

In 15 (11%) patients Dmin and in 11 (8%) patients Dmax were located within DIL-imaging, respectively. No significant differences in Dmean values delivered to PG, SPG and DIL-imaging (*p* > 0.05) were observed. The delivered Dmin and Dmax had significant differences between the three volumes, respectively (*p* < 0.05). The detailed characteristics of the dose parameter values for PG, SPG and DIL-imaging are listed in Table 1. Additionally, we tested the correlation between the three dose parameters in the respective three volumes (Additional file 2: Table S2). A weaker correlation between the Dmin values compared to the Dmean and Dmax values, respectively, was observed.

**Table 1:** Dose parameters

	Median, Mean (range)			
	PG	SPG	DIL-imaging	*p* value (Friedman test)
Dmean, Gy	75.5, 75 (65.9–79.8)	75.5, 75 (66–79.8)	75.3, 75 (65.7–79.9)	*p* = 0.296
Dmin, Gy	72, 70.6 (49.7–77.7)	72, 70.7 (49.7–77.7)	73.2, 72.5 (53.7–78.3)	*p* < 0.001
Dmax, Gy	77.8, 77.7 (68.4–82.3)	77.7, 77.7 (68.4–82.3)	77, 76.7 (67.3–82)	*p* < 0.001

Nonparametric Friedman test was used to evaluate the differences between the calculated dose-volume parameters. Post-hoc analyses using Wilcoxon matched-pairs signed rank test showed that the maximum dose was significantly lower in DIL-imaging compared to PG and SPG, whereas the minimum dose was significantly higher in DIL-imaging compared to PG and SPG (*p* < 0.05)

### Outcome

After a median follow-up time of 45 months (range: 14–116 months) 22 of 138 patients (16%) experienced a biochemical failure according to Phoenix criteria. Median PSA level at the time of recurrence was 4.8 ng/ml (range: 2.3–14.9 ng/ml). In 10 of 22 patients with BR (46%) the location of recurrence was detected based on PET or MRI and in 6 patients (27%) a local recurrence in the prostate was suspected. Visual assessment of the imaging data showed that the PCa lesion before EBRT and at appearance of BR had a high spatial overlap in 5 of these 6 patients. At the time of last evaluation 125 of 138 patients (91%) were alive and 2 deceased due to PCa.

### Impact of dose parameters on BRFS

**Fig. 2:**
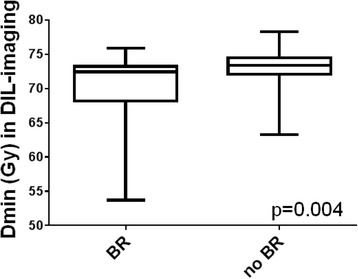
Comparison of Dmin values in patients with and without BR. Dmin was significant (*p* < 0.004) lower in patients with BR (72.4 Gy, range: 53.7–75.9 Gy) than in patients without BR (73.4 Gy, range: 63.3–78.3 Gy). Mann-Whitney test was used for comparison

Cut-off dose parameters for further analyses were determined: Dmean (tested range: 73.5–77.3 Gy) in all volumes was not a significant predictor for BRFS (*p* > 0.05). Dmin (tested range: 70–75.2 Gy) in PG and SPG had no significant impact on BRFS (p > 0.05), whereas Dmin (cut off value 70.6 Gy) in DIL-imaging was an independent prognostic factor for BRFS (HR = 0.39, *p* = 0.036) in multivariate analysis. Dmin was significant (*p* < 0.004) lower in patients with BR (72.4 Gy, range: 53.7–75.9 Gy) than in patients without BR (73.4 Gy, range: 63.3–78.3 Gy) (Fig. 2). In all three volumes multivariate analyses showed that Dmax (tested range: 75–79.8 Gy) was an independent risk factor with HR of 0.31–0.32 (*p* < 0.01) for DIL-imaging, SPG and PG, respectively. 101 of 110 patients (92%) with a Dmax ≥76 Gy in SPG had a Dmin ≥70.6 Gy in DIL-imaging. A summary of the uni- and multivariate Cox regression analyses considering BRFS is given in Tables 2 and 3.

**Table 2:** Cox-regression analyses considering BRFS

	*p* value
Patient and treatment related parameters	
iPSA in ng/ml (< 10, 10–20, > 20)	0.314
Gleason score in biopsy (< 7, 7, or > 7)	**0.018**
clinical T stage (2a + b, 2c, 3)	**0.031**
Volume DIL-imaging (continuous)	**0.034**
Age in years (continious)	0.773
Usage of ADT	0.223
Prescription dose in Gy (continious)	0.374
RT technique (3D, IMRT)	0.271

Results of the Cox regression analyses for the influence of different patient, treatment and dose parameters on the BRFS. Cut-off values for respective dose parameters were determined by an explorative analysis. None of the Dmin values were significant in PG and SPG, respectively. None of the binarized Dmean values were significant in any of the three volumes. Gleason score and cT stage were not significant (*p* < 0.05) in any of the multivariate analyses including dose parameters. Abbreviations: *CI* confidence interval, *HR* hazard ratio. Bold = values with *p* < 0.05

**Table 3:** Cox-regression analyses considering BRFS

Univariate Cox regression	*p* value	Multivariate Cox regression (with Gleason and cT stage)	HR (95 CI)	*p* value
Dose parameters				
Dmin DIL-imaging in Gy		Dmin DIL-imaging in Gy		
(< 70.6, ≥70.6)	**0.023**	(< 70.6, ≥70.6)	**0.039 (0.2–0.9)**	**0.036**
Dmax DIL-imaging in Gy		Dmax DIL-imaging in Gy		
(< 75.8, ≥75.8)	**0.013**	(< 75.8, ≥75.8)	**0.31 (0.1–0.7)**	**0.009**
Dmax SPG in Gy		Dmax SPG in Gy		
(< 76, ≥76)	**0.006**	(< 76, ≥76)	**0.32 (0.14–0.8)**	**0.009**
Dmax PG in Gy		Dmax PG in Gy		
(< 76, ≥76)	**0.006**	(< 76, ≥76)	**0.32 (0.14–0.8)**	**0.009**

Results of the Cox regression analyses for the influence of different patient, treatment and dose parameters on the BRFS. Cut-off values for respective dose parameters were determined by an explorative analysis. None of the Dmin values were significant in PG and SPG, respectively. None of the binarized Dmean values were significant in any of the three volumes. Gleason score and cT stage were not significant (*p* < 0.05) in any of the multivariate analyses including dose parameters. Abbreviations: *CI* confidence interval, *HR* hazard ratio. Bold = values with *p* < 0.05

**Fig. 3:**
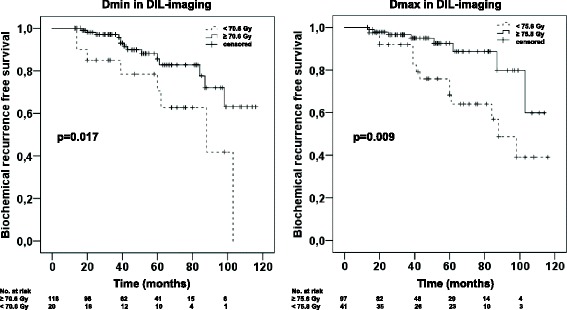
Kaplan-Meier curves for BRFS. Statistical comparison with Log-rank test revealed *p* < 0.017 and *p* < 0.009 when tested on Dmin (cut-off value 70.6 Gy) and Dmax (cut-off value 75.8 Gy), respectively

Kaplan-Meier curves on the impact of Dmin (70.6 Gy) and Dmax (75.8 Gy) applied to DIL-imaging for BRFS are shown in Fig. 3.

## Discussion

Several studies proposed the existence of a DIL as a driver of the metastatic and recurrent PCa post EBRT and concepts to detect and escalate RT dose to the DIL are under investigation [19, 20]. In this retrospective analysis we hypothesized that PSMA PET/CT and mpMRI are appropriate to localize the DIL (DIL-imaging) and that consequently, the dose parameters for DIL-imaging should correlate with the outcome in patients with PCa after EBRT. Our patients underwent conventional EBRT which aims at delivering a homogeneous dose to the entire prostatic gland without considering the localization of DIL-imaging. This explains why no significant differences between the Dmean values for all three volumes were observed and why Dmax was located only in 8% of the patients in DIL-imaging in in our study.

Dmin (cut-off: 70.6 Gy) in DIL-imaging influenced BRFS in multivariate analysis, whereas Dmin (range: 70–75.2 Gy) in PG and SPG had no impact on BRFS. Furthermore, median Dmin in DIL-imaging was significantly lower in patients with BR than in patients without BR. These findings support the theory of DIL since only an under dosage of clinically significant areas may lead to BR, whereas lower doses in non-DIL tissue had no impact on BRFS. Multivariate Cox regression showed a significant influence of Dmax within all three considered volumes for BRFS. The influence of Dmax in DIL-imaging may be explained by an increased killing of radio resistant PCa cells which may comprise tumour initiating features [21] or by possibly enhanced activation of the immune system [22]. The influence of Dmax in non-DIL-imaging tissue may be explained by the very high correlation between Dmax values in all three volumes. Furthermore, 92% of the patients with Dmin ≥70.6 Gy in DIL-imaging also had a Dmax ≥76 Gy in SPG.

In our study the binarized Dmean values (range: 73.5–77.3 Gy) in the respective three volumes had no significant impact on BRFS. However, several studies proved that the RT dose delivered on the entire prostatic gland has an impact on BRFS after primary EBRT for PCa [23, 24]. It should be mentioned, that no separate analysis of the dose distribution within DIL-imaging was performed in these studies. In our collective high correlations (rho> 0.7, *p* < 0.01) between Dmean values in PG and Dmax/Dmin values within DIL-imaging were calculated. What appears to be a significant influence of Dmean and Dmax in SPG/PG might therefore again be proof of the significance of Dmin and Dmax in DIL-imaging on BRFS**.**

Surely our observations need validation by future studies including more patients, longer follow-up and more dose parameters (e.g. D2%, D98% or Dmedian) for their analyses. However, our data provide evidence, that the delineation of the intraprostatic tumour using PSMA PET and mpMRI information should be performed routinely prior to RT of PCa in order to avoid under dosage and to possibly escalate the RT dose in these areas. Since there is no evidence of how to define patient populations in which one of the two imaging modalities performs better, the DIL-definition should preferably be performed based on combined PSMA PET and mpMRI information. Future work may also address which RT doses should be given to SPG since it still remains unclear if a dose ≥76 Gy on SPG is really needed in all patients. Keeping in mind that not the entire intraprostatic PCa amount is visible on mpMRI and PSMA PET dose de-escalation may possibly increase the risk for BR. On the other hand previous analyses [3–5] and this study showed that local recurrent disease after EBRT mostly occurred at the side of the primary tumour. Furthermore, our study indicated that the RT dose within the imaging defined DIL may be more crucial for the outcome after EBRT than the dose applied to SPG. To address this important question, RT planning studies should simulate de-escalation strategies while considering the dose distribution within the co-registered histological information [20].

This retrospective study has several limitations. The treatment protocols (e.g. ADT duration, RT technique) and the follow-up procedure are not identical for all patients. Thus, our results need validation, preferably by a prospective trial. A further shortcoming is the relatively short period of follow-up. Persistent testosterone suppression after adjuvant ADT might have an impact on PSA levels [25]. However, the median ADT duration in our cohort was 7 months. A longer follow-up would also enable the evaluation of other relevant endpoints like PCa-specific survival or overall survival. Eiber et al. [8] and our group [7] published on comparing mpMRI and PSMA PET/CT with histopathology after prostatectomy, both reporting a good sensitivity and specificity for mpMRI and PSMA PET/C, individually. However, the combined usage of both modalities achieved the highest sensitivity, indicating that they may offer complementary information. Most of the patients in our cohort (74%) had mpMRI only, thus an underestimation of the total PCa amount may have occured. On the other hand, several studies postulated that even if mpMRI may not detect the entire PCa tissue, it is able to detect a clinically sufficient amount of the tumour [26, 27]. For this study we supposed that mpMRI and PSMA PET/CT are equivalent in DIL-definition and we did not analyse them separately.

Another issue of this study is the uncertainty in registration of PET/CT, mpMRI and planning CT images (e.g. due different rectum and bladder fillings during imaging). To minimize geometrical errors we used automatic 3D matching tools and performed a manual re-adjustment if necessary. The insertion of intraprostatic fiducial markers visible in MRI and CT images would facilitate this process by enabling landmark based registration techniques. In this study the implantation of intraprostatic fiducial markers (94% of the patients) was performed 2–3 weeks before the planning CT and mostly after the MRI and PET scans. However, using daily image-guidance based on the markers we accounted for inter-fractional movement. Nevertheless, we were not able to account for intra-fractional movement and possible shifts of the target regions during RT. By implementing real-time tracking systems [28] or by using brachytherapy [29, 30] possible strategies to solve this problem have already been proposed.

## Conclusions

This study showed that the dose distribution within DILs defined by mpMRI and/or PSMA PET imaging are independent risk factors for biochemical failure after primary EBRT in patients with PCa. These findings support the implementation of modern imaging for DIL detection and may be considered in RT treatment planning to avoid an under dosage or to escalate the RT dose in these areas. Further validation in larger patient cohorts with longer follow-up should be warranted.

## Additional files

Additional file 1: **Table S1.** Patient characteristics. The detailed characteristics of the study cohort are listed. Abbreviation: *n* = number of patients. (PDF 88 kb)

Additional file 2: **Table S2.** Spearman’s rho test. Correlation between the three dose parameters in the respective three volumes was analyzed. A weaker correlation between the Dmin values compared to the Dmean and Dmax values, respectively, was observed. Spearman’s rho values are listed. The respective *p* values were all < 0.001. (PDF 135 kb)

## Abbreviations

ADC: Apparent diffusion coefficient
ADT: Androgen deprivation therapy
BR: Biochemical recurrence
BRFS: Biochemical recurrence free survival
cbCT: Cone-beam CT
CT: Computed tomography
CTV: Clinical target volume
DCE: Dynamic contrast-enhanced
DIL: Dominant intraprostatic lesions
Dmax: Maximum dose
Dmean: Mean dose
Dmin: Minimum dose
DVH: Dose volume histogram
DWI: Diffusion weighted images
EBRT: External beam radiation therapy
GTV-MRI: GTV based on mpMRI
GTV-PET: Gross tumour volume according to PET information
Gy: Gray
HR: Hazard ratio
IGRT: Image-guided RT
IMRT: Intensity-modulated RT
mpMRI: Multiparametric MRI
MRI: Magnetic resonance imaging
PCa: Prostate cancer
PET/CT: Positron emission tomography/ computed tomography
PG: Prostatic gland
PI-RADs v2: Prostate Imaging - Reporting and Data System version 2
PSA: Prostate specific antigen
PSMA: Prostate-specific membran antigen
PTV: Planning target volume
RT: Radiotherapy
SPG: Non-PCa prostate tissue
SUVmax: Maximum standardized uptake value
T2w-TSE: T2-weighted fast spin echo

## References

[1] Cooper CS, Eeles R, Wedge DC (2015). Analysis of the genetic phylogeny of multifocal prostate cancer identifies multiple independent clonal expansions in neoplastic and morphologically normal prostate tissue. Nat Genet.

[2] Haffner MC, Mosbruger T, Esopi DM (2013). Tracking the clonal origin of lethal prostate cancer. J Clin Invest.

[3] Arrayeh E, Westphalen AC, Kurhanewicz J (2012). Does local recurrence of prostate Cancer after radiation therapy occur at the site of primary tumor? Results of a longitudinal MRI and MRSI study. Int J Radiat Oncol.

[4] Pucar D, Hricak H, Shukla-Dave A (2007). Clinically significant prostate cancer local recurrence after radiation therapy occurs at the site of primary tumor: magnetic resonance imaging and step-section pathology evidence. Int J Radiat Oncol.

[5] Mendez LC, Ravi A, Chung H, et al. Pattern of relapse and dose received by the recurrent intraprostatic nodule in low- to intermediate-risk prostate cancer treated with single fraction 19 Gy high dose-rate brachytherapy. Brachytherapy. 2018;17(2):291-7. 10.1016/j.brachy.2017.10.001 29137956

[6] de Rooij M, Hamoen EH, Futterer JJ, Barentsz JO, Rovers MM (2014). Accuracy of multiparametric MRI for prostate cancer detection: a meta-analysis. AJR Am J Roentgenol.

[7] Zamboglou C, Drendel V, Jilg CA (2017). Comparison of 68Ga-HBED-CC PSMA-PET/CT and multiparametric MRI for gross tumour volume detection in patients with primary prostate cancer based on slice by slice comparison with histopathology. Theranostics.

[8] Eiber M, Weirich G, Holzapfel K (2016). Simultaneous 68Ga-PSMA HBED-CC PET/MRI improves the localization of primary prostate Cancer. Eur Urol.

[9] Fendler WP, Schmidt DF, Wenter V (2016). 68Ga-PSMA PET/CT detects the location and extent of primary prostate Cancer. J Nucl Med.

[10] Viani GA, Stefano EJ, Afonso SL (2009). Higher-than-conventional radiation doses in localized prostate cancer treatment: a meta-analysis of randomized, controlled trials. Int J Radiat Oncol Biol Phys.

[11] Martinez AA, Gonzalez J, Ye H (2011). Dose escalation improves Cancer-related events at 10 years for intermediate- and high-risk prostate Cancer patients treated with Hypofractionated high-dose-rate boost and external beam radiotherapy. Int J Radiat Oncol.

[12] Quivrin M, Loffroy R, Cormier L (2015). Multiparametric MRI and post implant CT-based dosimetry after prostate brachytherapy with iodine seeds: the higher the dose to the dominant index lesion, the lower the PSA bounce. Radiother Oncol.

[13] Zamboglou C, Wieser G, Hennies S (2016). MRI versus (68)Ga-PSMA PET/CT for gross tumour volume delineation in radiation treatment planning of primary prostate cancer. Eur J Nucl Med Mol Imaging.

[14] Eder M, Neels O, Muller M (2014). Novel preclinical and radiopharmaceutical aspects of [68Ga]Ga-PSMA-HBED-CC: a new PET tracer for imaging of prostate Cancer. Pharmaceuticals.

[15] Zamboglou C, Schiller F, Fechter T (2016). (68)Ga-HBED-CC-PSMA PET/CT versus histopathology in primary localized prostate Cancer: a voxel-wise comparison. Theranostics.

[16] Weinreb JC, Barentsz JO, Choyke PL (2016). PI-RADS prostate imaging - reporting and data system: 2015, version 2. Eur Urol.

[17] D'Amico AV, Chen MH, Roehl KA, Catalona WJ (2004). Preoperative PSA velocity and the risk of death from prostate cancer after radical prostatectomy. New Engl J Med.

[18] Roach M, Hanks G, Thames H (2006). Defining biochemical failure following radiotherapy with or without hormonal therapy in men with clinically localized prostate cancer: recommendations of the RTOG-ASTRO Phoenix consensus conference. Int J Radiat Oncol.

[19] Bauman G, Haider M, Van der Heide UA, Menard C (2013). Boosting imaging defined dominant prostatic tumors: a systematic review. Radiother Oncol.

[20] Zamboglou C, Sachpazidis I, Koubar K (2017). Evaluation of intensity modulated radiation therapy dose painting for localized prostate cancer using 68Ga-HBED-CC PSMA-PET/CT: a planning study based on histopathology reference. Radiother Oncol.

[21] Cojoc M, Peitzsch C, Kurth I (2015). Aldehyde dehydrogenase is regulated by beta-catenin/TCF and promotes Radioresistance in prostate Cancer progenitor cells. Cancer Res.

[22] Nesslinger NJ, Sahota RA, Stone B (2007). Standard treatments induce antigen-specific immune responses in prostate cancer. Clin Cancer Res.

[23] Pollack A, Zagars GK, Starkschall G (2002). Prostate cancer radiation dose response: results of the M. D. Anderson phase III randomized trial. Int J Radiat Oncol Biol Phys.

[24] Zelefsky MJ, Yamada Y, Fuks Z (2008). Long-term results of conformal radiotherapy for prostate cancer: impact of dose escalation on biochemical tumor control and distant metastases-free survival outcomes. Int J Radiat Oncol Biol Phys.

[25] D'Amico AV, Chen MH, Renshaw AA, Loffredo M, Kantoff PW (2009). Interval to testosterone recovery after hormonal therapy for prostate cancer and risk of death. Int J Radiat Oncol Biol Phys.

[26] Cash H, Maxeiner A, Stephan C (2016). The detection of significant prostate cancer is correlated with the prostate imaging reporting and data system (PI-RADS) in MRI/transrectal ultrasound fusion biopsy. World J Urol.

[27] Yuan Q, Costa DN, Senegas J (2017). Quantitative diffusion-weighted imaging and dynamic contrast-enhanced characterization of the index lesion with multiparametric MRI in prostate Cancer patients. J Magn Reson Imaging.

[28] Willoughby TR, Kupelian PA, Pouliot J (2006). Target localization and real-time tracking using the calypso 4D localization system in patients with localized prostate cancer. Int J Radiat Oncol.

[29] Zamboglou C, Rischke HC, Meyer PT (2016). Single fraction multimodal image guided focal salvage high-dose-rate brachytherapy for recurrent prostate cancer. J Contemp Brachytherapy.

[30] Gomez-Iturriaga A, Casquero F, Urresola A (2016). Dose escalation to dominant intraprostatic lesions with MRI-transrectal ultrasound fusion high-dose-rate prostate brachytherapy. Prospective phase II trial. Radiother Oncol.

