# Supplementary material for: The dose distribution in dominant intraprostatic tumour lesions defined by multiparametric MRI and PSMA PET/CT correlates with the outcome in patients treated with primary radiation therapy for prostate cancer
Source: Radiat Oncol. 2018 Apr 12;13:65. doi: 10.1186/s13014-018-1014-1 (PMC5898009; doi:10.1186/s13014-018-1014-1)
Supplement: Supplementary file 1 — Table S1. Patient characteristics. The detailed characteristics of the study cohort are listed. Abbreviation: n = number of patients. (PDF 88 kb) [file 13014_2018_1014_MOESM1_ESM.pdf]

Additional table 1. Patient characteristics

Median age in years (range)	74 (56-85)
Median initial PSA in ng/ml (range)	10.3 (3.1-47)
Biopsy Gleason score, n (%)	
6	19 (14)
7a	49 (36)
7b	43 (31)
8	17 (12)
9	10 (7)
10	0
Clinical T stage, n (%)	
2a	20 (14)
2b	24 (17)
2c	55 (40)
3a	30 (22)
3b	9 (7)
D'Amico risk group, n (%)	
low-risk	2 (1)
intermediate-risk	33 (24)
high-risk	103 (75)
Median, mean Volume, ml (range)	
PG	49.4, 53.2 (21.9-187.6)
SPG	45.1, 48.2 (17.6-177)
DIL-imaging	3.6, 5 (0.3-38)

Abbreviation: n = number of patients
