# Supplementary material for: The dose distribution in dominant intraprostatic tumour lesions defined by multiparametric MRI and PSMA PET/CT correlates with the outcome in patients treated with primary radiation therapy for prostate cancer
Source: Radiat Oncol. 2018 Apr 12;13:65. doi: 10.1186/s13014-018-1014-1 (PMC5898009; doi:10.1186/s13014-018-1014-1)
Supplement: Supplementary file 2 — Table S2. Spearman’s rho test. Correlation between the three dose parameters in the respective three volumes was analyzed. A weaker correlation between the Dmin values compared to the Dmean and Dmax values, respectively, was observed. Spearman’s rho values are listed. The respective p values were all < 0.001. (PDF 135 kb) [file 13014_2018_1014_MOESM2_ESM.pdf]

Additional table 2. Spearman's rho test

	DIL-imaging with SPG	DIL-imaging with PG	PG with SPG
Dmean	0.93	0.94	1
Dmax	0.89	0.91	0.99
Dmin	0.72	0.73	1

Spearman's rho values are listed. The respective p values were all < 0.001.
